# Therapy duration and improvement of ventricular function in *de novo* heart failure: the Heart Failure Optimization study

**DOI:** 10.1093/eurheartj/ehae334

**Published:** 2024-06-12

**Authors:** Christian Veltmann, David Duncker, Michael Doering, Siva Gummadi, Michael Robertson, Thomas Wittlinger, Byron J Colley, Christian Perings, Orvar Jonsson, Johann Bauersachs, Robert Sanchez, Lars S Maier

**Affiliations:** Department of Cardiology and Angiology, Hannover Medical School, Carl-Neuberg-Str. 1, 30625 Hannover, Germany; Heart Center Bremen, Electrophysiology Bremen, Senator-Wessling-Str. 1, 28277 Bremen, Germany; Department of Cardiology and Angiology, Hannover Medical School, Carl-Neuberg-Str. 1, 30625 Hannover, Germany; Heart Center Leipzig, University of Leipzig, Leipzig, Germany; Department of Cardiology, CVI of Central Florida, Ocala, FL, USA; Department of Cardiology, Trinity Medical, WNY, Buffalo, NY, USA; Department of Cardiology, Asklepios Harzklinik Goslar, Goslar, Germany; Department of Cardiology, Jackson Heart Clinic, Jackson, MS, USA; Department of Cardiology, Katholisches Klinikum Luenen, Luenen, Germany; Department of Cardiology, Sanford Cardiovascular Institute, Sioux Falls, SD, USA; Department of Cardiology and Angiology, Hannover Medical School, Carl-Neuberg-Str. 1, 30625 Hannover, Germany; Department of Cardiology, HCA Florida Heart Institute, St. Petersburg, FL, USA; Department of Internal Medicine II, University Hospital Regensburg, Regensburg, Germany

**Keywords:** Heart failure, Wearable cardioverter-defibrillator, Guideline-recommended medical therapy

## Abstract

**Background and Aims:**

In patients with *de novo* heart failure with reduced ejection fraction (HFrEF), improvement of left ventricular ejection fraction (LVEF) is expected to occur when started on guideline-recommended medical therapy. However, improvement may not be completed within 90 days.

**Methods:**

Patients with HFrEF and LVEF ≤ 35% prescribed a wearable cardioverter-defibrillator between 2017 and 2022 from 68 sites were enrolled, starting with a registry phase for 3 months and followed by a study phase up to 1 year. The primary endpoints were LVEF improvement > 35% between Days 90 and 180 following guideline-recommended medical therapy initiation and the percentage of target dose reached at Days 90 and 180.

**Results:**

A total of 598 patients with *de novo* HFrEF [59 years (interquartile range 51–68), 27% female] entered the study phase. During the first 180 days, a significant increase in dosage of beta-blockers, renin–angiotensin system inhibitors, and mineralocorticoid receptor antagonists was observed (*P* < .001). At Day 90, 46% [95% confidence interval (CI) 41%–50%] of study phase patients had LVEF improvement > 35%; 46% (95% CI 40%–52%) of those with persistently low LVEF at Day 90 had LVEF improvement > 35% by Day 180, increasing the total rate of improvement > 35% to 68% (95% CI 63%–72%). In 392 patients followed for 360 days, improvement > 35% was observed in 77% (95% CI 72%–81%) of the patients. Until Day 90, sustained ventricular tachyarrhythmias were observed in 24 wearable cardioverter-defibrillator carriers (1.8%). After 90 days, no sustained ventricular tachyarrhythmia occurred in wearable cardioverter-defibrillator carriers.

**Conclusions:**

Continuous optimization of guideline-recommended medical therapy for at least 180 days in HFrEF is associated with additional LVEF improvement > 35%, allowing for better decision-making regarding preventive implantable cardioverter-defibrillator therapy.

## Structured Graphical Abstract

**Structured Graphical Abstract:**
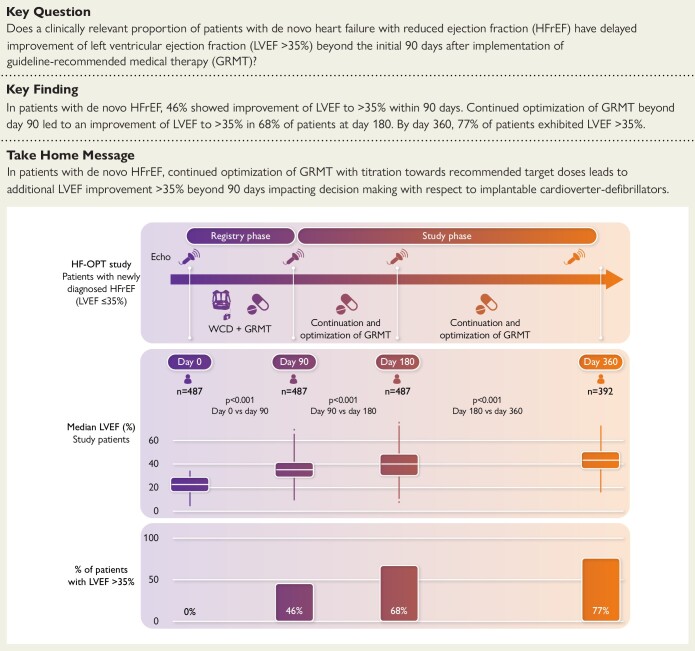
Summary of design and results of Heart Failure Optimization study. Median left ventricular ejection fraction and percentage of patients with left ventricular ejection fraction > 35% at baseline, Days 90, 180, and 360. GRMT, guideline-recommended medical therapy; HFrEF, heart failure with reduced ejection fraction; LVEF, left ventricular ejection fraction; WCD, wearable cardioverter-defibrillator.

**See the editorial comment for this article ‘Guideline-recommended medical therapy for de novo heart failure with reduced ejection fraction: be patient waiting for reverse remodelling’, by J.A. Borovac, https://doi.org/10.1093/eurheartj/ehae400.**

## Introduction

In patients with *de novo* heart failure (HF) with reduced ejection fraction (HFrEF), state-of-the-art interventional and medical therapy results in a considerable rate of improvement of left ventricular ejection fraction (LVEF).^1–3^

Despite newer recommendations for early implementation and up-titration of guideline-recommended medical therapy (GRMT), optimization of GRMT may require weeks to months to ‘up titrate’ dosages of HF medication in real-world practice.^4–6^ Recent data continue to show under-dosing over the first several months post-diagnosis.^7–9^ Within 90 days after diagnosis, optimized and stable pharmacological therapy is often not achieved.^10^ Thus, left ventricular reverse remodelling is likely not completed in this short period. Findings of the PROLONG study show that 33% of patients with non-improved low LVEF at 90 days may indeed improve LVEF to >35% by continuing optimization of GRMT beyond the first 3 months.^1^ These findings impact indication and timing for primary preventive implantable cardioverter-defibrillator (ICD) implantation, which is mainly based on LVEF ≤ 35% despite established optimal GRMT for a minimum of 90 days.^4,11^

To our knowledge, LVEF improvement has never been prospectively evaluated in a large multi-centre study with set time points for follow-up and repeated LVEF measurements, regardless of device management, and detailed HF medication data collection. Thus, the Heart Failure Optimization (HF-OPT) study was designed to evaluate continued left ventricular reverse remodelling beyond 90 days in patients with *de novo* HFrEF.

## Methods

### Study design and oversight

The rationale and design of the HF-OPT study are described elsewhere.^12^ In brief, HF-OPT is a multi-centre prospective observational study of patients with *de novo* HFrEF to test the hypothesis that additional LVEF improvement occurs between 90 and 180 days as GRMT is advanced (ClinicalTrials.gov identifier: NCT03016754). This study is being conducted according to international standards of good clinical practice and has been approved by the medical ethics committees. All patients gave written informed consent. The study was sponsored by ZOLL Medical, Pittsburgh, PA, USA.

The HF-OPT study consisted of two phases: the registry phase: *de novo* HF, wearable cardioverter-defibrillator (WCD) prescription, and start of GRMT; and the study phase: further optimization of GRMT and evaluation of primary and secondary endpoints. The study started in March 2017 and ended in May 2022.

### Study patients

Eligibility requirements for inclusion in the registry phase were age ≥ 18 years, *de novo* HF (LVEF ≤ 35%) for ischaemic (ICM) or non-ischaemic cardiomyopathy (NICM) and prescription of the WCD.^13^ Eligibility for the study phase included completion of the registry phase and prescription of the WCD for 90 ± 14 days unless they received an ICD prior to 90 days of WCD. Of note, LVEF at Day 90 did not need to be ≤35% to be included in the study.

### Study procedures

After inclusion in HF-OPT, the registry phase started for 90 ± 14 days from hospital discharge followed by the study phase (Days 91–360). Continued WCD prescription after the first 90 days was not a requirement for the study phase, and medical and device therapy was left to the discretion of the physician.

Left ventricular ejection fraction measurements were performed at 0, 90 (±14), 180 (±14), and 360 (±14) days after WCD start. For this study, LVEF improvement > 35% was defined as an increase in LVEF > 35%.

Wearable cardioverter-defibrillator device records were interrogated to determine compliance with use, defibrillation events, and arrhythmia detection. Wearable cardioverter-defibrillator electrocardiographic (ECG) automatic recordings were also collected. All shocked events, deaths, and ECG recordings were adjudicated by a clinical events committee.

### Guideline-recommended medical therapy

Guideline-recommended medical therapy was defined by the use of guideline-recommended therapies at the time of the study, such as HF treatments with any renin–angiotensin system inhibitor and beta-blockers (BBs)^4,11^ (see Supplementary data online, *Table S1*). Mineralocorticoid receptor antagonists (MRAs) should be included along with angiotensin-converting enzyme inhibitor (ACE-I) and BB treatments. All medications should be titrated to reach evidence-based doses (or the maximally tolerated levels). Adherence to GRMT is defined as reaching target evidence-based doses (or the maximally tolerated levels) of these treatments. According to guidelines, additional HF medications included angiotensin receptor–neprilysin inhibitor (ARNI), *I_f_*-channel inhibitor, or digitalis glycosides in selected patients. Missing doses of GRMT were imputed with the last dose available.

### Study outcomes

The primary endpoints of this study were to assess (i) the rate of improvement of LVEF > 35% between Days 90 and 180 in patients with *de novo* HFrEF while being started and optimized on GRMT and (ii) the degree to which GRMT had been achieved. Secondary endpoints included mortality and WCD-specific data like wear-time, compliance, brady- and tachyarrhythmias, and treatments during the registry and study phase.

### Statistical analysis

Based on prior observational data of WCD users, we expected ∼40% to have LVEF improvement > 35% during the first 90 days.^1,14^ We hypothesized that an additional 5% of all patients from the index hospital discharge (regardless of device therapy) would experience LVEF improvement between Days 90 and 180 as optimal GRMT is achieved.

For analysis of the primary endpoint, LVEF at Days 90 and 180 was compared. The lower bound of 95% confidence interval (CI) was tested against 5%. The enrolment of 1400 patients prescribed with WCD (registry phase) was estimated to provide 600 patients eligible for the study at Day 90.

Descriptive statistics were used to report major clinical characteristics, rates of improvement > 35%, and frequency of events. These data were analysed using *t*-tests and Wilcoxon test for continuous variables and χ^2^ tests for categorical variables. The time to event for each patient was defined from the first day of WCD wear to the first event day. Univariable and multivariable logistic regression analysis was used to examine the association of clinical variables with LVEF improvement > 35%. For multivariable logistic regression analysis, variables that are significantly correlated with LVEF > 35% at Day 180 in univariate analysis were included. Multivariable analyses were conducted in patients with complete data sets (*n* = 487).

Cumulative survival was calculated using the Kaplan–Meier method. All analyses were performed using R software (version 4.1.2). A *P*-value of <.05 was considered statistically significant.

## Results

### Patients

**Figure 1:**
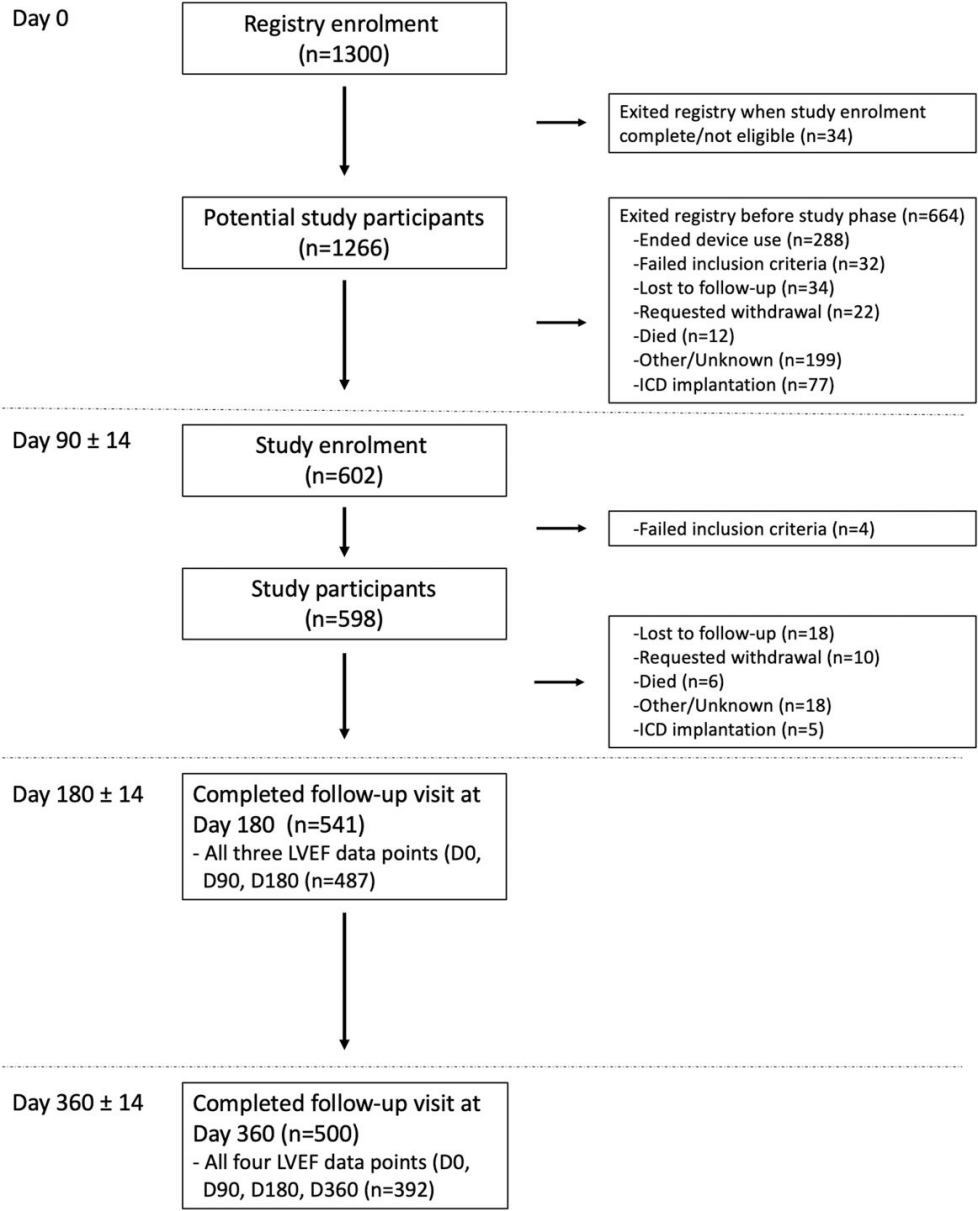
CONSORT flow diagram with disposition of patients in Heart Failure Optimization study. ICD, implantable cardioverter-defibrillator; LVEF, left ventricular ejection fraction

Overall, 1300 patients with *de novo* HFrEF were enrolled in the registry. Patients were included at 68 sites in the USA (*n* = 662), Germany (*n* = 575), France (*n* = 46), and Austria (*n* = 17). When study enrolment met the target number, enrolment stopped at 602 patients and 30 registry patients were exited from the registry phase, prior to study phase eligibility. Another four enrolled patients were later determined to be ineligible for failure to meet the inclusion/exclusion criteria. Thus, 1266 registry patients comprised the denominator, or ‘pool’, of potential patients for the study in the ‘run-in’ phase, and 598 study phase patients were eligible for analysis (*Figure 1*). Not advancing to the study phase was primarily due to termination of the WCD use before Day 90 visit (*n* = 329), mostly after an early echocardiogram prior to 90 days displayed recovered LVEF (*n* = 209) or an ICD or cardiac resynchronization therapy (CRT) device was implanted early (*n* = 41) and the WCD was discontinued. Clinical characteristics of the study phase patients are listed in *Table 1*. The median age was 59 years [interquartile range (IQR) 51–68], 27% were female, and the median LVEF during the index hospitalization was 23 (IQR 18–28). New York Heart Association (NYHA) functional class was II or III in 81% of patients with known NYHA class. NICM was the predominant aetiology in 58% of HF patients. Regarding medical therapies, at diagnosis of *de novo* HFrEF, a total of 17 patients (1.3%) received inotropics or vasopressors intravenously; 95% of patients were initially prescribed BB; 92% an ACE-I, angiotensin receptor blockers (ARBs), or ARNI; 60% a MRA; and 5% ivabradine. Therapy of BB plus an ACE-I, ARB, or ARNI was prescribed in 88% at discharge, while initial therapy of BB, an ACE-I, ARB, or ARNI, and an MRA was prescribed in 56%.

**Table 1:** Clinical characteristics of the study phase patients

	Study (*n* = 598)
Age, years, median (IQR)	59 (51–68)
Male sex, *n*, (%)	434 (73)
Ethnicity^a^, *n* (%)	
Caucasian	485 (81.1)
American Indian or Alaskan native	4 (.7)
Hispanic	14 (2.3)
Black or African American	74 (12.4)
Asian	5 (.8)
Other	1 (.2)
No response	19 (3.1)
BMI (kg/m^2^), median (IQR)	28.6 (24.9–32.9)
Initial heart rate, b.p.m, median (IQR)	80 (70–92)
Initial respiration rate, breaths/min, median (IQR)	16 (16–18)
Systolic blood pressure, mmHg, median (IQR)	117 (106–132)
History of HTN, *n* (%)	383 (64)
History of diabetes, *n* (%)	162 (27)
History of COPD, *n* (%)	71 (12)
History of TIA/CVA, *n* (%)	41 (7)
History of CKD, *n* (%)	65 (11)
NYHA class (index hospitalization), *n* (%)	
I	14 (2.3)
II	115 (19.2)
III	224 (37.5)
IV	65 (10.9)
Not documented	180 (30.1)
Predominant HF aetiology, *n* (%)	
ICM	252 (42)
NICM	346 (58)
Predominant NICM aetiology	
Idiopathic	122 (20)
Hypertensive	48 (8.0)
Tachycardia-induced	35 (5.8)
Myocarditis	38 (6.4)
Alcohol-induced	21 (3.5)
Valvular	10 (1.7)
Other Substance-induced	8 (1.3)
Cocaine-induced	5 (.84)
Post-partum	9 (1.5)
Other	73 (12)
Initial LVEF during index hospitalization, %, median (IQR)	23 (18–28)
History of pacemaker, *n* (%)	8 (1.3)
History of ICD, *n* (%)	1 (.17)
History of MI, *n* (%)	128 (21.4)
History of CABG, *n* (%)	50 (8.4)
History of PCI, *n* (%)	133 (22)
History of SCA, *n* (%)	18 (3.0)
History of arrhythmia in past year^a^, *n* (%)	263 (44)
Atrial flutter	24 (4.0)
Atrial fibrillation	113 (19)
Sinus tachycardia	85 (14)
Supraventricular tachycardia	13 (2.2)
Ventricular tachycardia	13 (2.2)
Ventricular fibrillation	1 (.2)
Non-sustained ventricular tachycardia	28 (4.7)
Sinus bradycardia	10 (1.7)
First-degree AV block	6 (1.0)
Second-degree AV block (type I)	1 (.17)
Second-degree AV block (type II)	1 (.17)
Third-degree AV block	1 (.17)
Paced rhythm	3 (.50)
History of angina in past year	148 (25)
History of syncope	32 (5.5)
Initial HF medications	
RAS inhibitor	552 (92)
ACE-I/ARB	406 (68)
ARNI	149 (25)
Beta-blockers	570 (95)
Mineralocorticoid receptor antagonists	359 (60)
Cardiac glycosides	39 (6.5)
Ivabradine	31 (5.2)

ACE-I, angiotensin-converting enzyme inhibitor; ARB, angiotensin receptor blocker; ARNI, Angiotensin receptor–neprilysin inhibitor; AV, atrioventricular; BB, beta-blocker; BMI, body mass index; CABG, coronary artery bypass graft; CKD, chronic kidney disease; COPD, chronic obstructive pulmonary disease; HF, heart failure; HTN, hypertension; ICD, implantable cardioverter-defibrillator; ICM, ischaemic cardiomyopathy; IQR, interquartile range; LVEF, left ventricular ejection fraction; MI, myocardial infarction; NICM, non-ischaemic cardiomyopathy; NYHA, New York Heart Association; PCI, Percutaneous Coronary Intervention; SCA, sudden cardiac arrest; TIA/CVA, transient ischaemic attack/cerebrovascular accident.

^a^More than one selection could be made.

### Left ventricular ejection fraction: registry phase

**Figure 2:**
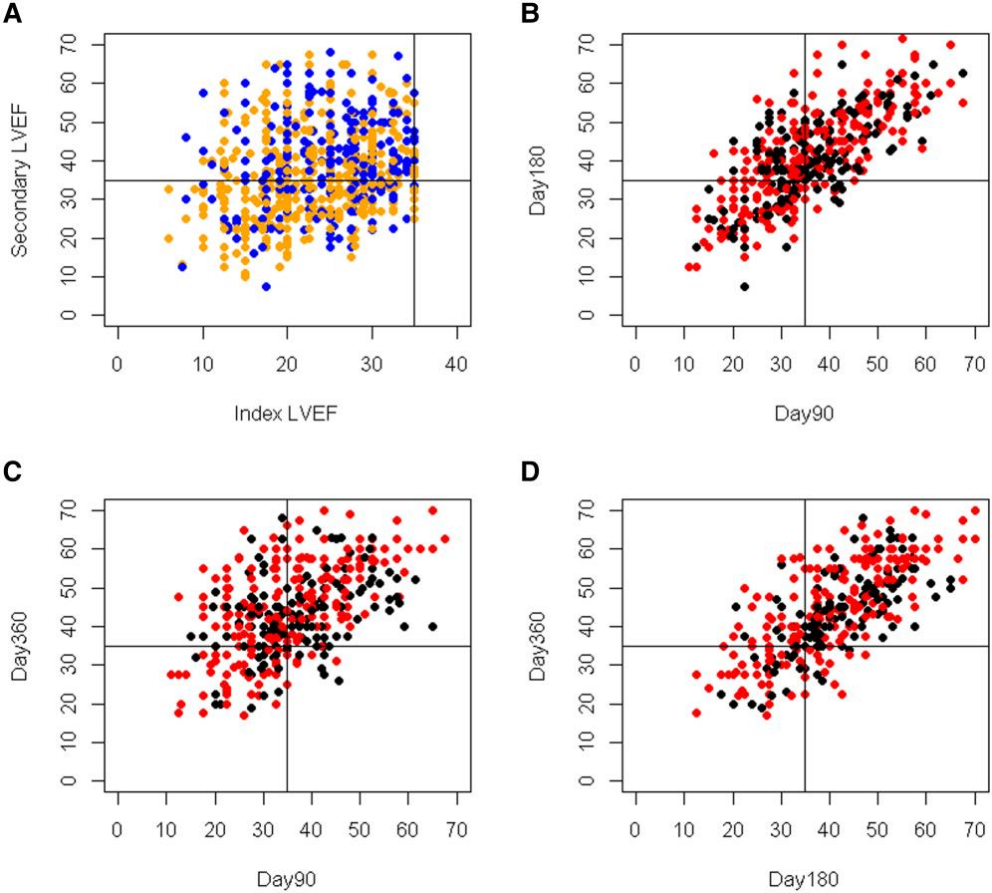
Scatter plots depicting paired data of left ventricular ejection fraction. (*A*) Index left ventricular ejection fraction vs. left ventricular ejection fraction at first follow-up visit (90 days or wearable cardioverter-defibrillator discontinuation; ‘secondary left ventricular ejection fraction’). (*B*) Left ventricular ejection fraction at Day 90 vs. Day 180. (*C*) Left ventricular ejection fraction at Day 90 vs. Day 360. (*D*) Left ventricular ejection fraction at Day 180 vs. Day 360. Blue dots: ‘Registry only’ patients. Orange dots: ‘Study only’ patients. Black dots: Patients with ischaemic cardiomyopathy. Red dots: Patients with non-ischaemic cardiomyopathy. LVEF, left ventricular ejection fraction

Out of 1300 patients, 903 registry phase patients had LVEF data collected from both the index and first follow-up visit [median 87 days (IQR 77–96)]. Median LVEF was 24% (IQR 19%–29%) at the index hospitalization and improved to 38% (IQR 30%–45%) at the first follow-up (*P* < .001). At the first follow-up, 55% (499/903) of patients displayed an improvement of LVEF > 35% (*Figure 2*).

### Left ventricular ejection fraction: study phase

For the analysis of LVEF during the study phase, only those with all three LVEF values (Days 0, 90, and 180) were analysed (*n* = 487). Median LVEF improved from 23% (IQR 18%–28%) at index to 34% (IQR 28%–43%) at Day 90 and to 40% (IQR 33%–48%) at Day 180 (*P* < .001) (*Structured Graphical Abstract*).

At Day 90, 222 (46%) of study phase patients had early improvement > 35%. Only 14 out of these 222 patients (6%) deteriorated again by Day 180 (see Supplementary data online, *Figure S1*). These patients had a median LVEF of 41% (IQR 38%–42%) at Day 90 followed by a decline [median 33% (IQR 32%–34%)] at Day 180.

Of 265 (54%) study phase patients with persistently low LVEF at Day 90, 122 (46%) showed LVEF improvement > 35% by Day 180.

Univariable analysis of all baseline variables (see Supplementary data online, *Table S2*) showed that the following variables were significantly associated with LVEF improvement > 35% by Day 180: systolic blood pressure (BP) [median 120 (IQR 110–136) vs. 113 (IQR 103–124) mmHg], index LVEF [median 23% (IQR 19–29) vs. 20% (IQR 15–25)], and atrial fibrillation in the previous year (23.0% vs. 13.4%) (*Table 2*). Sudden cardiac arrest (SCA) (1.2% vs. 5.7%) and non-sustained ventricular tachycardia (VT) in the previous year (3.6% vs. 8.3%) were associated with no improvement of LVEF > 35%. At multivariable analysis, systolic BP [odds ratio (OR) 1.02, 95% CI 1.01–1.03], initial LVEF (OR 1.07, 95% CI 1.04–1.10), and atrial fibrillation (OR 1.98, 95% CI 1.16–3.49) were predictors of LVEF improvement > 35% by Day 180, whereas SCA (OR 0.14, 95% CI 0.03–0.47) was associated with no improvement of LVEF > 35% (*Table 2*).

**Table 2:** Predictors of improvement of left ventricular ejection fraction > 35% at Day 180

	Univariable analysis			Multivariable analysis		
	OR	95% CI	*P*-value	OR	95% CI	*P*-value
Systolic blood pressure (per mmHg)	1.02	1.01–1.03	<.001	1.02	1.01–1.03	<.001
Initial LVEF during index hospitalization (per %)	1.07	1.04–1.10	<.001	1.07	1.04–1.10	<.001
History of SCA	0.20	0.05–0.63	.01	.14	0.03–0.47	.002
Atrial fibrillation in previous year	1.94	1.16–3.35	.01	1.98	1.16–3.49	.01
NSVT in previous year	0.42	0.18–0.94	.04	.53	0.23–1.25	.15

CI, confidence interval; LVEF, left ventricular ejection fraction; NSVT, non-sustained ventricular tachycardia; OR, odds ratio; SCA, sudden cardiac arrest.

**Figure 3:**
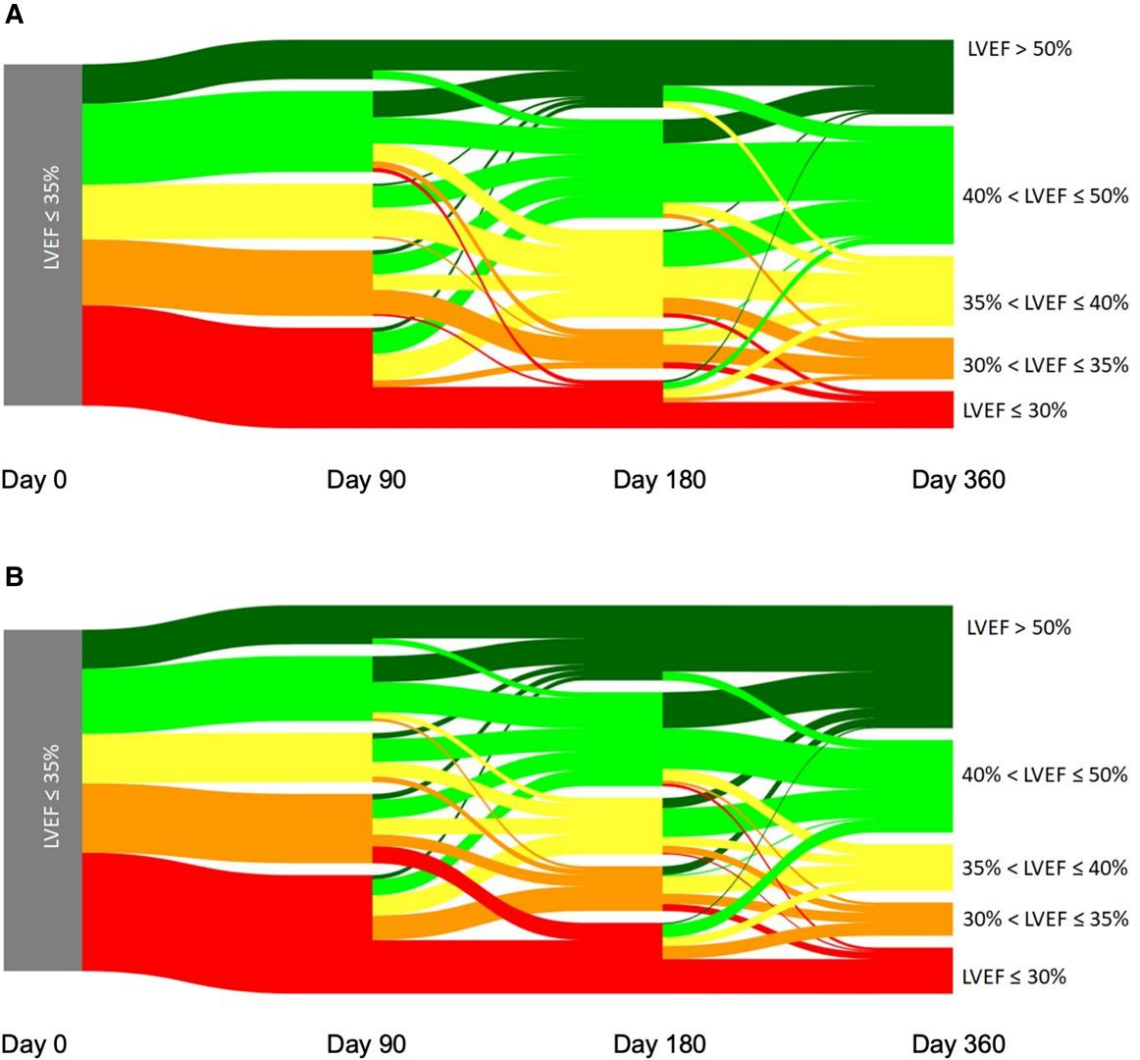
Sankey plot of individual changes in left ventricular ejection fraction from Day 0 to Day 360 in patients with (*A*) ischaemic cardiomyopathy (*n* = 236) and (*B*) non-ischaemic cardiomyopathy (*n* = 156). LVEF, left ventricular ejection fraction

A total of 392 patients were echocardiographically followed until Day 360. In this subset of patients, further improvement of LVEF > 35% was observed in 77% (95% CI 72%–81%) (see Supplementary data online, *Figure S1* and *Table S3*). With respect to aetiology, patients with ICM or NICM had different baseline characteristics (see Supplementary data online, *Table S4*). Patients with NICM tend to recover LVEF > 50% more often than patients with ICM; however, improvement > 35% was not different at Day 90, 180, and 360 (*Figure 3*; Supplementary data online, *Tables S5–S8*).

### Medication prescription rates and degree of target dosage

This analysis was done in those study phase patients with LVEF data at all three time points (*n* = 487) and medications available. Beta-blocker, ACE-I/ARB/ARNI, and MRA were prescribed in 95%, 92%, and 60% at index, 96%, 94%, and 62% at Day 90, and 97%, 94% and 62% at Day 180, respectively. Angiotensin receptor–neprilysin inhibitor was prescribed in 21%, 38%, and 42% at index, Days 90 and 180, respectively. Beta-blocker and an ACE-I/ARB or ARNI was prescribed in 92% at Day 180, while all three classes (BB, ACE-I/ARB, or ARNI, and MRA) at Day 180 were prescribed in 60%.

During the first 180 days, an increase in dosage of BB, renin–angiotensin system inhibitors, and MRA was observed (*P* < .001 for all three). Those who were prescribed target doses of ACE-I/ARB/ARNI and MRA experienced higher rates of improvement > 35% than those not on target doses at Day 90 (ACE-I/ARB/ARNI, 53% vs. 42%, *P* = .02; and MRA, 52% vs. 41%, *P* = .02). By Day 180, target doses of all drug classes were associated with LVEF improvement > 35% at that time point (*P* = .048) (*Table 3*). In those 45 patients (9%) on target doses of all drug classes at day 180, 89% had LVEF improvement > 35%, vs. only 60% of those on <100% target of all drugs (see Supplementary data online, *Table S9*). Patients reaching target doses of BB and ACE-I/ARB/ARNI (7%) showed LVEF improvement > 35% in 72% of the cases. The prescription rate of loop diuretics decreased from 75% of the patients at Day 0 to 67% at Day 180 (see Supplementary data online, *Table S10*).

**Table 3:** Guideline-recommended medical therapy target levels correlate with left ventricular ejection fraction improvement in study phase patients

		Dose unknown	Dose ≥ target level		Dose < target level		*P*-value
			LVEF ≤ 35% at Day 180	LVEF > 35% at Day 180	LVEF ≤ 35% at Day 180	LVEF > 35% at Day 180	
BB	Day 90	2	52 (48%)	57 (52%)	212 (56%)	164 (44%)	.14
BB	Day 180	0	30 (25%)	92 (75%)	127 (35%)	238 (65%)	.048
ACE-I/ARB/ARNI	Day 90	2	75 (47%)	86 (53%)	188 (58%)	136 (42%)	.02
ACE-I/ARB/ARNI	Day 180	2	48 (24%)	150 (76%)	107 (37%)	180 (63%)	.003
MRA	Day 90	0	107 (48%)	114 (52%)	158 (59%)	108 (41%)	.02
MRA	Day 180	0	56 (26%)	161 (74%)	101 (37%)	169 (63%)	.01

Analysis for 487 subjects for the association of medication at Day 90 and Day 180 with LVEF at Day 180.

ACE-I, angiotensin-converting enzyme inhibitor; ARB, angiotensin receptor blocker; ARNI, angiotensin receptor–neprilysin inhibitor; BB, beta-blocker; MRA, mineralocorticoid receptor antagonist.

### Wearable cardioverter-defibrillator data

Of the 1300 patients (registry and study), 1289 wore the WCD on at least 1 day for ≥15 min. Of these 1289, the median wearing time was 22.4 h/day. Median WCD wear time in all registry-only patients with a minimum 1 day of wear for ≥15 min (*n* = 687) was 22.5 h/day worn during the first 90 days. All study phase patients (*n* = 598) wore the WCD for at least 1 day, with a median of 22.7 h/day.

During WCD use, 24 registry patients of the 1300 (1.8%) experienced 34 sustained VT/ventricular fibrillation (VF) events within the first 90 days. Of these, seven patients were appropriately treated by the WCD (3.2 patients treated per 100 patient-years of WCD use). The remaining patients were not shocked due to the use of the response buttons to abort shock delivery. During the first 90 days, the overall sustained VT/VF event rate was 15.5 events per 100 patient-years. Between Days 90 and 180 in one WCD carrier, a non-sustained VT was observed.

Non-WCD defibrillations were also reported for five patients: two patients received automatic external defibrillation during the registry phase after reported SCA while not wearing the WCD (50% survival) and three patients reported appropriate ICD shocks during the study phase (100% survival). Electrocardiograms were not available for adjudication.

Overall, nine patients experienced inappropriate WCD shocks (*n* = 5 to atrial fibrillation, *n* = 4 due to over-sensing). There was one serious adverse device event due to skin irritation.

### Mortality

For the study population (*n* = 598), cumulative survival at Days 180, 270, and 360 was 99.2%, 98.5%, and 97.7%, respectively.

During the whole study period, there were 28 deaths. Half of the deaths (*n* = 14) were adjudicated as cardiovascular. Eleven patients (0.8%) died during the first 90 days (registry phase). Of those, nine patients died from cardiovascular cause [five from HF, two other/unknown, one asystole, one in-hospital sudden cardiac death (SCD)]. Seventeen patients (2.8%) died after Day 90, with five for cardiovascular reasons: one asystole (documented by the WCD at the time of death), two SCDs (both not wearing a WCD, one patient implanted with an ICD), one HF, and one in-hospital post-myocardial infarction.

## Discussion

Heart Failure Optimization study is the first prospective study analysing the improvement of LVEF > 35% beyond 90 days in patients with *de novo* HFrEF and severely reduced LVEF under continuous optimization of medical therapy. The main findings of HF-OPT are as follows: (i) at Day 90 after *de novo* HFrEF, 46% of patients improved LVEF > 35%; (ii) an additional 46% of those with LVEF ≤ 35% at 90 days had LVEF improvement > 35% by Day 180 up to a total of 77% of patients with an LVEF > 35% at Day 360; (iii) HF GRMT including BB, MRA, and ACE-I/ARB/ARNI was continuously optimized and associated with LVEF improvement > 35%, but target dosage was reached only in the minority of patients; (iv) one-third (32%) of patients with no LVEF improvement up to Day 90 displayed LVEF improvement > 35% by Day 180; and (v) ventricular tachyarrhythmias occurred mainly in the first 90 days after diagnosis, while after 90 days, only one non-sustained VT occurred.

### Left ventricular ejection fraction

Current pharmacological and interventional therapies induce reverse remodelling after *de novo* HF leading to different degrees of LVEF improvement > 35% depending on HF aetiology. Within the first 90 days after diagnosis, the potential of improvement > 35% is highest with the maximal gain in LVEF. However, within these 90 days, reverse remodelling is not completed in a considerable number of patients.^1,15^ Although LVEF is measured at HF diagnosis, and usually at some follow-up period, it is unknown how often LVEF should be determined and what ‘improvement > 35%’ of left ventricular dysfunction means in terms of outcomes. Most such analyses have only been done in patients with chronic HF, and not with mandatory LVEF re-assessment at set time points.^16^ Patients with improved LVEF seem to have better outcomes than those with no improvement.^17–19^ The PROVE-HF study enrolled patients with chronic HFrEF, and LVEF was assessed at 6 and 12 months. Left ventricular ejection fraction improvement > 35% was documented in 32% and 62% at 6 and 12 months after starting sacubitril/valsartan.^20^ We saw a slightly higher improvement > 35% in our patients with *de novo* HFrEF at 90, 180, and 360 days (46%, 68%, and 77%, respectively). Notably, LVEF improvement > 35% was not always sustained, as 6% patients with initial improvement deteriorated again between 90 and 180 days. The PROLONG study also showed a further increase in LVEF beyond Day 90, especially in those with non-ischaemic aetiologies.^1,21^ Additionally, patients without an ICD recommendation after prolonged WCD prescription did not experience SCD during follow-up.^22^

In the current HF-OPT study, we were able to show that 46% of patients with an LVEF ≤ 35% at Day 90 improve to an LVEF > 35% at Day 180. In a subset of 392 patients, we were able to show that improvement of LVEF > 35% continued to 77% at Day 360. This indicates that the process of reverse remodelling in both ischaemic and non-ischaemic aetiologies is not completed 3 months after diagnosis. Although LVEF improvement > 35% was not different between patients with ICM and NICM, patients with NICM tended to recover in LVEF > 50% more often following GRMT than patients with ICM. Following CRT, response with respect to LVEF improvement is also better in patients with NICM.^4^ Lower degree of ventricular substrate or ischaemic scar burden might be the cause for both findings.

Besides a higher initial LVEF, predictors for improvement LVEF > 35% were higher BP at baseline, which leave room for reaching target dosages of HF drugs with antihypertensive effects. Interestingly, atrial fibrillation was also associated with LVEF improvement. This finding most likely refers to consequent rhythm or rate control after diagnosis of *de novo* HF.

Apart from medication and dosage, time seems to play an important role in recovery from HFrEF. These findings impact decision-making for or against primary prophylactic ICD implantation. Currently, the implantation of an ICD is indicated in patients with an LVEF ≤ 35% at the earliest of 90 days under GRMT.^4,23,24^ This cannot be achieved within 90 days after diagnosis. Probably, even after 180 days, this is hardly achievable. The decision for ICD implantation should, therefore, be taken if the patient is medically optimized and LVEF is stable with no further improvement under optimized GRMT, which may take more time than 90 or even 180 days. Following the establishment of optimal pharmacologic HF therapy, additional non-invasive risk assessment can be performed to support decision-making of primary prophylactic ICD implantation.^25,26^

### Guideline-recommended medical therapy

Implementation and optimization of GRMT after *de novo* HFrEF is crucial for reverse remodelling. For several classes, beneficial effects with respect to morbidity and mortality have been shown.^11,27^ The more drugs prescribed and the closer to the recommended target dose these medications are up-titrated, the higher the benefit.^5^ An approach of modelling an accelerated application of combination GRMT for HF showed that the faster this implementation period is, the higher the survival.^28^ Recently, the STRONG-HF study in patients admitted for acute HF demonstrated all-cause death and HF readmission after 180 days were reduced by an intensive treatment strategy of rapid up-titration of GRMT and close follow-up compared with usual care.^29^

Problems with achieving GRMT appear to be hampered by a lack of physician and patient adherence. A retrospective study of Medicare patients showed a very low rate of GRMT being achieved in the 90 days before ICD implantation.^30^ Only 61.1% had filled their prescriptions for a BB plus an ACE-I or ARB at least once during the 90 days before ICD implantation, and only 28.3% had a supply for ≥80% of the 90 days. Interestingly, those with the shortest duration of HF and the most recent evaluation of LVEF were the least likely to receive GRMT. Death within 1 year occurred more frequently in those patients not receiving any GRMT even after adjusting for patient characteristics, HF severity, and comorbidities. The QUALIFY registry also showed that physician adherence to GRMT is associated with better outcomes in HFrEF.^31^ Results from the current HF-OPT study are similar in that target doses of BB and ACE-I/ARB/ARNI were achieved in <50% of patients, even by 180 days. Regarding the at first sight rather low use of ARNI in HF-OPT, it has to be mentioned that at the time of the study, ACE-I was the first-line treatment recommendation in *de novo* HFrEF with a switch to ARNI considered during follow-up after target dosages of ACE-I had been achieved.

Further, those patients having LVEF improvement > 35% at 90 and 180 days were more often taking optimal GRMT compared with those without LVEF improvement > 35%. Thus, early initiation and rapid up-titration seem to improve outcomes.^32^ In HF-OPT, ARNI prescription increased between Days 90 and 180. The change from ACE-I/ARB to ARNI was recommended by the guidelines if ACE-I/ARB was up-titrated. This might have impacted further LVEF improvement after Day 90 positively. To date, the ARNI is recommended as first-line therapy after *de novo* HFrEF. One may speculate that improvement of LVEF may occur earlier and to a larger extent with ARNI as first-line treatment. However, even with HF management using BB/ARB-ACE-I/MRA (although target doses are not reached in most patients), a high rate of LVEF improvement > 35% can be reached.

### Ventricular tachyarrhythmias

The majority of sustained ventricular tachyarrhythmias occurred within the first 90 days after diagnosis of HFrEF (34 events in 1.8% of the patients). Beyond the first 90 days, only one event of a non-sustained VT was observed. These findings are consistent with previous reports on ventricular arrhythmias after *de novo* HF. WEARIT-II observed a 3-month overall 2% cumulative probability of sustained VT/VF, which is similar to our study.^14^ In MADIT-RIT, the rate of appropriate ICD shocks was three appropriate shocks per 100 patient-years.^33^ Progress in pharmacologic HF management has demonstrated positive outcomes for HFrEF patients over the past decade.^34^ However, these achievements did not lead to reduction in mortality or arrhythmic risk during the early phase as achieving therapeutic dosages and conveyance of clinical benefits take several months. Especially in this very early phase when medication is initiated, but far away from optimized, the WCD plays a role in protecting patients from SCD.^22,35,36^ On the other side, the risk for ventricular arrhythmias in the later phase beyond Day 90 is low. This tributes to the early impact of GRMT in the early phase after initiation.^29,37,38^ According to current guidelines, all four prognostically relevant drugs should be initiated and up-titrated rapidly.^5^ Earlier reverse remodelling is expected by following this protocol and consecutively a lower incidence of ventricular arrhythmias. According to the results of the present study, the prescription of a WCD should be based on an individual case decision.

### Limitations

This study may have bias due to the WCD wear requirement. Patients were required to have the WCD for 90 days prior to study enrolment. Also, while the study enrolled *de novo* HF patient post-hospitalization, we cannot exclude that some patients may have had longer-lasting low LVEF without symptomatic HF in the past. This may underestimate the potential LVEF improvement > 35%. A limitation of HF-OPT is the large number of dropouts during the registry phase. This is mainly caused by preliminary termination of WCD wearing. However, after entering the study phase, attrition was much lower, allowing to analyse the primary endpoint in a total of 487 patients. Although hypothesis generating, this study was not powered to measure SCD risk rates or survival differences between groups.

Finally and maybe most importantly, sodium-glucose co-transporter 2 inhibitors were not yet part of the standard of care for HF patients, and ARNI has been slowly adopted, so our results may even underestimate the potential LVEF improvement > 35% of GRMT at Day 180 and even Day 360.

## Conclusions

First, initiation and optimization of GRMT beyond Day 90 after diagnosis is mandatory to further improve LVEF. Second, evaluation of ICD implantation should not be done before day 180 after diagnosis. Even beyond Day 180 continued, improvement was observed. Third, in the early phase after *de novo* diagnosis of HFrEF with severely reduced LVEF, VT/VF occurrence is highest and WCD should be considered.

## Supplementary Material

ehae334_Supplementary_Data

## Data Availability

The data underlying this article will be shared on reasonable request to the corresponding author.

## Pre-registered Clinical Trial Number

NCT03016754
