# Supplementary material for: Therapy duration and improvement of ventricular function in de novo heart failure: the Heart Failure Optimization study
Source: Eur Heart J. 2024 Jun 12;45(30):2771–81. doi: 10.1093/eurheartj/ehae334 (PMC11313580; doi:10.1093/eurheartj/ehae334)
Supplement: ehae334_Supplementary_Data [file ehae334_supplementary_data.docx]

**Supplementary data**

Supplementary table 1: Target doses of GRMT and corresponding number of patients.

| **Type of therapy** | **Drug** | **Initial dose** | **Target maximum dose** | **Patients on medication at day 0** | **Patients on medication at day 90** | **Patients on medication at day 180** |
| --- | --- | --- | --- | --- | --- | --- |
| ACE inhibitor | Captopril | 6.25 mg 3 times daily | 50 mg 3 times daily | 0 | 0 | 0 |
|  | Enalapril | 2.5 mg twice daily | 10 mg twice daily | 2 | 3 | 3 |
|  | Lisinopril | 2.5 mg once daily | 20 mg once daily | 120 | 96 | 84 |
|  | Ramipril | 1.25 mg once daily | 10 mg once daily | 147 | 114 | 97 |
|  | Perindopril | 2 mg once daily | 8 mg once daily | 2 | 1 | 0 |
|  | Benazepril | 10 mg once daily | 40 mg once daily | 5 | 4 | 4 |
| ARB | Candesartan | 4 mg once daily | 32 mg once daily | 13 | 11 | 11 |
|  | Irbesartan | 75 mg once daily | 300 mg once daily | 4 | 3 | 3 |
|  | Telmisartan | 40 mg once daily | 80 mg once daily | 1 | 0 | 0 |
|  | Olmesartan | 10 mg once daily | 40 mg once daily | 2 | 1 | 1 |
|  | Valsartan | 20 mg twice daily | 160 mg twice daily | 17 | 14 | 16 |
|  | Losartan | 25 mg once daily | 50 mg once daily | 35 | 32 | 33 |
| ARNI | Sacubitril-valsartan | 49/51 mg twice daily | 97/103 mg twice daily | 102 | 183 | 206 |
| Beta-blocker | Carvedilol | 3.125 mg twice daily | 25 mg twice daily | 158 | 154 | 158 |
|  | Metoprolol succinate | 12.5 mg once daily | 200 mg once daily | 136 | 129 | 126 |
|  | Toprol-XL | 12.5 mg once daily | 200 mg once daily | 37 | 45 | 45 |
|  | Metoprolol | 12.5 mg once daily | 200 mg once daily | 129 | 136 | 138 |
|  | Bisoprolol | 1.25 mg once daily | 10 mg once daily | 129 | 136 | 138 |
|  | Atenolol | 50 mg once daily | 200 mg once daily | 1 | 0 | 0 |
|  | Labetalol | 100 mg twice daily | 400 mg once daily | 1 | 1 | 1 |
|  | Nebivolol | 1.25 mg once daily | 10 mg once daily | 3 | 5 | 7 |
|  | Propranolol | 40 mg twice daily | 120 twice daily | 0 | 0 | 0 |
| Mineralocorticoid receptor antagonist | Spironolactone | 12.5 mg daily | 25 mg daily | 39 | 37 | 36 |
|  | Eplerenone | 25 mg daily | 50 mg daily | 96 | 91 | 95 |

Any dose ranges or differences between the guidelines are represented here as a single number, with the lowest value chosen. Abbreviations: ACE: angiotensin-converting enzyme; ARB: angiotensin receptor blocker; ARNI: Angiontensin receptor neprilysin inhibitor; GRMT: Guideline recommended medical therapy.

Supplementary table 2: Results of the univariable analysis of baseline characteristics with respect to improvement of LVEF >35% at Day180.

|  | Day 180 LVEF ≤35 (n=157) | Day 180 LVEF >35 (n=330) | OR | 95% CI  Lower bound | 95% CI  Upper bound | p-value |
| --- | --- | --- | --- | --- | --- | --- |
| Age (years), median (IQR) | 59 (51-69) | 60 (52-68) | 1 | 0.99 | 1.01 | 0.96 |
| Gender, male n (%) | 112 (71.3%) | 240 (72.7%) | 0.93 | 0.61 | 1.43 | 0.75 |
| Race, n (%) |  |  |  |  |  |  |
| Caucasian | 126 (80.3%) | 271 (82.1%) | 1.13 | 0.69 | 1.82 | 0.62 |
| Hispanic | 4 (2.5%) | 8 (2.4%) | 0.95 | 0.29 | 3.61 | 0.94 |
| Black or African American | 20 (12.7%) | 37 (11.2%) | 0.87 | 0.49 | 1.57 | 0.62 |
| Asian | 2 (1.3%) | 3 (0.9%) | 0.71 | 0.12 | 5.44 | 0.71 |
| No response | 5 (3.2%) | 11 (3.3%) | 1.05 | 0.37 | 3.38 | 0.93 |
| BMI (kg/m^2^), median (IQR) | 29.2 (25.4-32.5) | 28.3 (24.6-32.9) | 0.99 | 0.97 | 1.02 | 0.55 |
| Initial heart rate (bpm), median (IQR) | 83 (74-95) | 80 (68-91) | 0.99 | 0.98 | 1 | 0.16 |
| Initial respiration rate (brpm), median (IQR) | 17 (16-18) | 16 (16-18) | 0.97 | 0.9 | 1.05 | 0.5 |
| Systolic blood pressure (mmHg), median (IQR) | 113 (103-124) | 120 (110-136) | 1.02 | 1.01 | 1.03 | <0.001 |
| History of HTN, n (%) | 88 (56.1%) | 215 (65.2%) | 1.47 | 0.99 | 2.16 | 0.053 |
| History of diabetes, n (%) | 46 (29.3%) | 78 (23.6%) | 0.75 | 0.49 | 1.15 | 0.18 |
| History of COPD, n (%) | 17 (10.8%) | 43 (13%) | 1.23 | 0.69 | 2.29 | 0.49 |
| History of TIA/CVA, n (%) | 10 (6.4%) | 24 (7.3%) | 1.15 | 0.55 | 2.58 | 0.72 |
| History of CKD, n (%) | 22 (14%) | 30 (9.1%) | 0.61 | 0.34 | 1.11 | 0.10 |
| NYHA class (index hospitalisation), n (%) |  |  |  |  |  |  |
| I | 3 (1.9%) | 9 (2.7%) |  |  |  |  |
| II | 25 (15.9%) | 64 (19.4%) | 0.85 | 0.18 | 3.13 | 0.82 |
| III | 66 (42%) | 122 (37%) | 0.62 | 0.13 | 2.15 | 0.48 |
| IV | 18 (11.5%) | 41 (12.4%) | 0.76 | 0.15 | 2.9 | 0.7 |
| Not available | 45 (28.7%) | 94 (28.5%) | 0.7 | 0.15 | 2.46 | 0.6 |
| Heart failure etiology, n (%) |  |  |  |  |  |  |
| ICM | 59 (37.6%) | 140 (42.4%) |  |  |  |  |
| NICM | 98 (62.4%) | 190 (57.6%) | 0.82 | 0.55 | 1.20 | 0.31 |
| Initial LVEF during index hospitalisation (%), median (IQR) | 20 (15-25) | 23 (19-29) | 1.07 | 1.04 | 1.11 | <0.001 |
| History of pacemaker, n (%) | 3 (1.9%) | 4 (1.2%) | 0.63 | 0.14 | 3.23 | 0.55 |
| History of MI, n (%) | 38 (24.2%) | 65 (19.7%) | 0.77 | 0.49 | 1.22 | 0.26 |
| History of CABG, n (%) | 11 (7%) | 24 (7.3%) | 1.04 | 0.51 | 2.26 | 0.92 |
| History of PCI, n (%) | 38 (24.2%) | 63 (19.1%) | 0.74 | 0.47 | 1.17 | 0.19 |
| History of SCA, n (%) | 9 (5.7%) | 4 (1.2%) | 0.2 | 0.05 | 0.63 | 0.01 |
| History of arrhythmia in previous year, n (%) | 63 (40.1%) | 158 (47.9%) | 1.37 | 0.93 | 2.02 | 0.11 |
| Atrial flutter | 5 (3.2%) | 12 (3.6%) | 1.15 | 0.42 | 3.66 | 0.8 |
| Atrial fibrillation | 21 (13.4%) | 76 (23%) | 1.94 | 1.16 | 3.35 | 0.01 |
| Sinus tachycardia | 25 (15.9%) | 47 (14.2%) | 0.88 | 0.52 | 1.5 | 0.63 |
| Supraventricular tachycardia | 2 (1.3%) | 9 (2.7%) | 2.17 | 0.55 | 14.36 | 0.33 |
| Ventricular tachycardia | 3 (1.9%) | 7 (2.1%) | 1.11 | 0.3 | 5.22 | 0.88 |
| Ventricular tachycardia (non- sustained) | 13 (8.3%) | 12 (3.6%) | 0.42 | 0.18 | 0.94 | 0.04 |
| Sinus bradycardia | 2 (1.3%) | 8 (2.4%) | 1.93 | 0.48 | 12.86 | 0.41 |
| History of angina, n (%) | 43 (27.4%) | 81 (24.5%) | 0.86 | 0.56 | 1.33 | 0.50 |
| History of syncope, n (%) | 11 (7%) | 14 (4.2%) | 0.59 | 0.26 | 1.36 | 0.2 |

* Variables with occurrence in less than 5 patients (race American indian or Alaskan, history of ICD, ventricular fibrillation, first degree AV block, type I second degree AV block, type II second degree AV block, third degree AV block, paced rhythm) were excluded from univariable analysis.

Abbreviations: AV: atrio-ventricular; BB: beta blocker; BMI: body mass index; BPM: Beats per minute; BRPM: Breaths per minute; CABG: Coronary artery bypass graft surgery; CKD: chronic kidney disease; COPD: Chronic obstructive pulmonary disease; HF: Heart failure; HTN: hypertension; ICD: implantable cardioverter-defibrillator; ICM: ischemic cardiomyopathy; IQR: interquartile range; MI: myocardial infarction; NICM: non-ischemic cardiomyopathy; NYHA: New-York heart association; PCI: Percutaneous Coronary Intervention; SCA: sudden cardiac arrest; TIA/CVA: Transient ischemic attack/Cerebrovascular accident.

Supplementary table 3: Proportion of patients with LVEF>35% from Day 0 to Day 360 in all patients.

|  | % patients with LVEF>35% (95% CI) | Median LVEF (IQR) | Patients (n) |
| --- | --- | --- | --- |
| Day 0 | 0% | 23 (18-28) | 487 |
| Day 90 | 46% (41%-50%) | 34 (28-43) | 487 |
| Day 180 | 68% (63%-72%) | 40 (33-48) | 487 |
| Day 360 | 77% (72%-81%) | 44 (37-53) | 392 |

Abbreviations: CI: confidence interval; IQR: Interquartile range; LVEF: Left ventricular ejection fraction.

Supplementary table 4: Baseline characteristics of patients with ischemic and non-ischemic cardiomyopathy.

|  | Ischemic cardiomyopathy  N=199 | Non-ischemic cardiomyopathy  N=288 | p-value |
| --- | --- | --- | --- |
| Age, median (IQR) | 61 (54-71) | 58 (49-67) | <0.001 |
| Gender, male n (%) | 157 (78.9%) | 195 (67.7%) | 0.009 |
| Ethnicity, n (%) | 22 (11.1%) | 68 (23.6%) |  |
| Caucasian | 177 (88.9%) | 220 (76.4%) | 0.001 |
| American Indian or Alaskan native | 0 (0%) | 2 (0.7%) | 0.647 |
| Hispanic | 2 (1%) | 10 (3.5%) | 0.153 |
| Black or African American | 12 (6%) | 45 (15.6%) | 0.002 |
| Asian | 2 (1%) | 3 (1%) | 1 |
| Other | 1 (0.5%) | 0 (0%) | 0.852 |
| No response | 6 (3%) | 10 (3.5%) | 0.984 |
| BMI (kg/m^2^), median (IQR) | 27.7 (24.3-32.3) | 29.3 (25.4-33.3) | 0.04 |
| Initial heart rate (BPM), median (IQR) | 80 (70-92) | 81 (71-94) | 0.7 |
| Initial respiration rate (BRPM), median (IQR) | 16 (16-18) | 16 (16-18) | 0.89 |
| Systolic blood pressure (mmHg), median (IQR) | 120 (110-138) | 116 (105-130) | 0.01 |
| History of HTN, n (%) | 139 (69.8%) | 164 (56.9%) | 0.005 |
| History of diabetes, n (%) | 61 (30.7%) | 63 (21.9%) | 0.038 |
| History of COPD, n (%) | 24 (12.1%) | 36 (12.5%) | 0.996 |
| History of TIA/CA, n (%) | 13 (6.5%) | 21 (7.3%) | 0.887 |
| History of CKD, n (%) | 29 (14.6%) | 23 (8%) | 0.03 |
| NYHA class (index hospitalisation), n (%) |  |  |  |
| I | 5 (2.5%) | 7 (2.4%) | 1 |
| II | 48 (24.1%) | 41 (14.2%) | 0.008 |
| III | 68 (34.2%) | 120 (41.7%) | 0.12 |
| IV | 25 (12.6%) | 34 (11.8%) | 0.91 |
| Not available | 53 (26.6%) | 86 (29.9%) | 0.51 |
| Initial LVEF during index hospitalisation (%), median (IQR) | 25 (20-29) | 21 (17.5-27.5) | <0.001 |
| History of pacemaker, n (%) | 2 (1%) | 5 (1.7%) | 0.78 |
| History of ICD, n (%) | 1 (0.5%) | 0 (0%) | 0.852 |
| History of MI, n (%) | 87 (43.7%) | 16 (5.6%) | <0.01 |
| History of CABG, n (%) | 30 (15.1%) | 5 (1.7%) | <0.01 |
| History of PCI, n (%) | 92 (46.2%) | 9 (3.1%) | <0.01 |
| History of SCA, n (%) | 10 (5%) | 3 (1%) | 0.017 |
| History of arrhythmia in previous year, n (%) | 74 (37.2%) | 147 (51%) | 0.003 |
| Atrial flutter | 6 (3%) | 11 (3.8%) | 0.823 |
| Atrial fibrillation | 36 (18.1%) | 61 (21.2%) | 0.469 |
| Sinus tachycardia | 21 (10.6%) | 51 (17.7%) | 0.04 |
| Supraventricular tachycardia | 4 (2%) | 7 (2.4%) | 1 |
| Ventricular tachycardia | 4 (2%) | 6 (2.1%) | 1 |
| Ventricular tachycardia (non-sustained) | 6 (3%) | 19 (6.6%) | 0.121 |
| Sinus bradycardia | 4 (2%) | 6 (2.1%) | 1 |
| 1^st^ AV block | 2 (1%) | 3 (1%) | 1 |
| 2^nd^ AV block Type I | 1 (0.5%) | 0 (0%) | 0.852 |
| 2^nd^ AV block Type II | 0 (0%) | 1 (0.3%) | 1 |
| 3^rd^ AV block | 1 (0.5%) | 0 (0%) | 0.852 |
| Paced rhythm | 0 (0%) | 3 (1%) | 0.392 |
| History of angina | 68 (34.2%) | 56 (19.4%) | <0.001 |
| History of syncope | 12 (6%) | 13 (4.5%) | 0.592 |

Abbreviations: AV: atrio-ventricular; BB: beta blocker; BMI: body mass index; BPM: Beats per minute; BRPM: Breaths per minute; CABG: Coronary artery bypass graft surgery; CKD: chronic kidney disease; COPD: Chronic obstructive pulmonary disease; HF: Heart failure; HTN: hypertension; ICD: implantable cardioverter-defibrillator; ICM: ischemic cardiomyopathy; IQR: interquartile range; MI: myocardial infarction; NICM: non-ischemic cardiomyopathy; NYHA: New-York heart association; PCI: Percutaneous Coronary Intervention; SCA: sudden cardiac arrest; TIA/CVA: Transient ischemic attack/Cerebrovascular accident.

Supplementary table 5: Development of LVEF from day 0 to day 360 according to LVEF classification for all patients (n=487).

|  | Day 90 | Day 180 | Day 360 |
| --- | --- | --- | --- |
| LVEF < 30%, (n (%)) | 170 (35) | 102 (21) | 49 (10) |
| 30% < LVEF ≤ 35%, (n (%)) | 95 (20) | 55 (11) | 42 (9) |
| 35% < LVEF ≤ 40%, (n (%)) | 67 (14) | 97 (20) | 64 (13) |
| 40% < LVEF ≤ 50%, (n (%)) | 101 (21) | 130 (27) | 118 (24) |
| LVEF > 50%, (n (%)) | 54 (11) | 103 (21) | 119 (24) |
| Not available, (n (%)) | 0 (0) | 0 (0) | 95 (20) |

Abbreviations: LVEF: Left ventricular ejection fraction

Supplementary table 6: Development of LVEF from day 0 to day 360 according to LVEF classification for patients with ischemic cardiomyopathy (n=199).

|  | Day 90 | Day 180 | Day 360 |
| --- | --- | --- | --- |
| LVEF < 30%, (n (%)) | 67 (34) | 38 (19) | 17 (9) |
| 30% < LVEF ≤ 35%, (n (%)) | 40 (20) | 21 (11) | 19 (10) |
| 35% < LVEF ≤ 40%, (n (%)) | 30 (15) | 48 (24) | 32 (16) |
| 40% < LVEF ≤ 50%, (n (%)) | 42 (21) | 57 (29) | 54 (27) |
| LVEF > 50%, (n (%)) | 20 (10) | 35 (18) | 34 (17) |
| Not available, (n (%)) | 0 (0) | 0 (0) | 43 (22) |

Abbreviations: LVEF: Left ventricular ejection fraction.

Supplementary table 7: Development of LVEF from day 0 to day 360 according to LVEF classification for patients with non-ischemic cardiomyopathy (n=288).

|  | Day 90 | Day 180 | Day 360 |
| --- | --- | --- | --- |
| LVEF < 30%, (n (%)) | 103 (36) | 64 (22) | 32 (11) |
| 30% < LVEF ≤ 35%, (n (%)) | 55 (19) | 34 (12) | 23 (8) |
| 35% < LVEF ≤ 40%, (n (%)) | 37 (13) | 49 (17) | 32 (11) |
| 40% < LVEF ≤ 50%, (n (%)) | 59 (20) | 73 (25) | 64 (22) |
| LVEF > 50%, (n (%)) | 34 (12) | 68 (24) | 85 (30) |
| Not available, (n (%)) | 0 (0) | 0 (0) | 52 (18) |

Abbreviations: LVEF: Left ventricular ejection fraction.

Supplementary table 8: LVEF from Day 0 to Day 360 for patients with ischemic (ICM) vs. non-ischemic (NICM) cardiomyopathy.

|  | Patients (n) | | % pts with LVEF > 35%  (% (CI)) | | | LVEF  (Median (IQR)) | | | Change of LVEF from Day 0 (median (IQR)) | | |
| --- | --- | --- | --- | --- | --- | --- | --- | --- | --- | --- | --- |
|  | ICM | NICM | ICM | NICM | P-value | ICM | NICM | P-value | ICM | NICM | P-value |
| Day 0 | 199 | 288 | - | - | - | 25  (20-29) | 22  (18-28) | 0.72 | - | - | - |
| Day 90 | 199 | 288 | 46%  (39-53%) | 45%  (39-51%) | 0.31 | 35  (29-43) | 33  (27-43) | 0.57 | 10  (4-18) | 11  (5-20) | 0.67 |
| Day 180 | 199 | 288 | 70%  (63-76%) | 66%  (60-71%) | 0.09 | 40  (33-47) | 40  (33-50) | 0.85 | 15  (8-22) | 20  (10-27) | 0.78 |
| Day 360 | 156 | 236 | 77%  (69-83%) | 77%  (71-82%) | 0.96 | 43  (37-49) | 45  (37-55) | 0.99 | 16  (10-25) | 24  (13-32) | 0.99 |

Abbreviations: CI: confidence interval; ICM: Ischemic cardiomyopathy; IQR: Interquartile range; LVEF: Left ventricular ejection fraction; NICM: Non-ischemic cardiomyopathy.

Supplementary table 9: Doses of GRMT and corresponding LVEF recovery at day 180.

| BB (% target) | ACEI/ARNI/ARB  (% target) | MRA (% target) | LVEF ≤35%  (n) | LVEF >35%  (n) | % patients LVEF>35 |
| --- | --- | --- | --- | --- | --- |
| <100 | <100 | <100 | 64 | 96 | 60 |
| <100 | <100 | ≥100 | 28 | 58 | 67 |
| <100 | ≥100 | <100 | 17 | 38 | 69 |
| <100 | ≥100 | ≥100 | 16 | 46 | 74 |
| <100 | NA | <100 | 1 | 0 | 0 |
| <100 | NA | ≥100 | 1 | 0 | 0 |
| ≥100 | <100 | <100 | 9 | 9 | 50 |
| ≥100 | <100 | ≥100 | 6 | 17 | 74 |
| ≥100 | ≥100 | <100 | 10 | 26 | 72 |
| ≥100 | ≥100 | ≥100 | 5 | 40 | 89 |

Abbreviations: ACE: angiotensin-converting enzyme; ARB: angiotensin receptor blocker; ARNI: Angiotensin receptor neprilysin inhibitor; BB: Beta-blocker; MRA: Mineralocorticoid receptor antagonist; GRMT: Guideline recommended medical therapy. LVEF: Left ventricular ejection fraction.

Supplementary table 10: Prescription of loop diuretics day 0, day 90 and day 180.

|  | Patients on  any loop diuretic | Patients on  Furosemide | Patients on Torasemide | Patients on Bumetanide |
| --- | --- | --- | --- | --- |
| Index | 363 (75%) | 197 (40%) | 158 (32%) | 8 (1%) |
| Day 90 | 346 (71%) | 176 (36%) | 163 (33%) | 7 (1%) |
| Day 180 | 327 (67%) | 168 (34%) | 152 (31%) | 7 (1%) |

Supplementary table 11: NYHA class of study patients at day 90 and day 180.

| NYHA class at day 90 (n (%))  I  II  III  IV  Not documented | 147 (25%)  360 (60%)  84 (14%)  3 (1%)  4 (1%) |
| --- | --- |
| NYHA class at day 180 (n (%))  I  II  III  IV  Not documented | 183 (31%)  294 (49%)  61 (10%)  2 (0%)  58 (10%) |

Abbreviations: NYHA: New-York Heart Association.

**Supplementary figures**

Supplementary figure 1: Sankey plot of individual changes in left ventricular ejection fraction (LVEF) in all patients from day 0 to day 360.


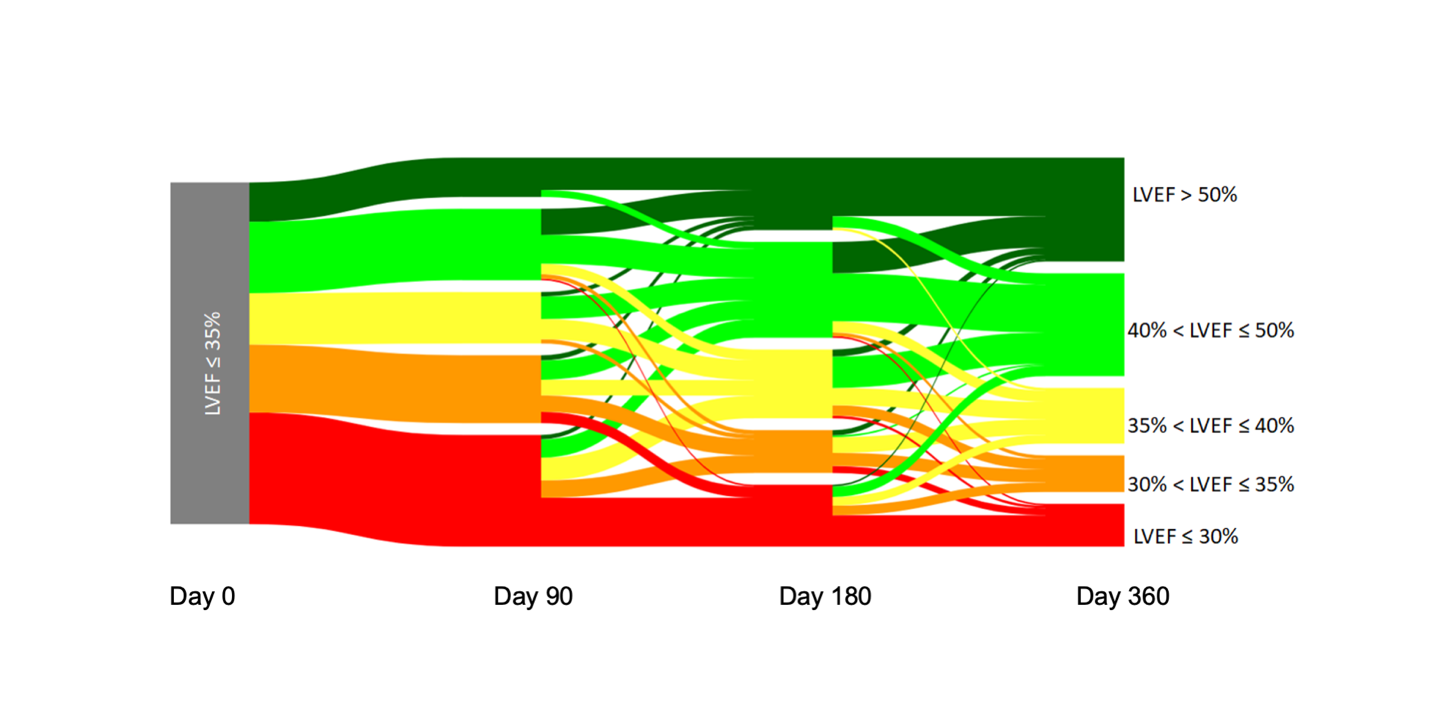


Statement: Impact of missing data on LVEF improvement analysis:

At day 90, 97% of study patients (583 out of 598) had LVEF measurement. 279 out of the 583

patients (48%) had LVEF >35%. This result is similar to 46% observed in 487 patients.

At day 180, 82% of study patients (490 out of 598) had EF measurement. LVEF at day 180 can be

imputed using Multiple Imputation by Chained Equations (mice). Based on imputed data, 67%

patients with LVEF >35% at day 180, similar to 68% observed in 487 patients.
